# A public health framework for reparations and generational healing in Haiti

**DOI:** 10.1371/journal.pgph.0004133

**Published:** 2025-11-04

**Authors:** Judite Blanc, Candice A. Sternberg, Anthony Q. Briggs, Ernest J. Barthélemy

**Affiliations:** 1 Department of Psychiatry and Behavioral Sciences, University of Miami Miller School of Medicine, Miami, Florida, United States of America; 2 Division of Infectious Disease, University of Miami Miller School of Medicine, Miami, Florida, United States of America; 3 Department of Neurology, Center for Cognitive Neurology, New York University Grossman School of Medicine, New York, New York, United States of America; 4 One Brooklyn Health/Brookdale University Hospital, Orthopedic Surgery and Public Health, Brooklyn, New York, United States of America; 5 SUNY Downstate Health Sciences University, Brooklyn, New York, United States of America; Institute of Public Health Bengaluru, INDIA

## Abstract

Amid the dismantling of state structures in Haiti, the first Black republic faces significant health disparities compared to its former colonial power, France. These disparities include lower life expectancy (64.8 vs. 82.3 years) and higher infant and maternal mortality rates. The situation is further exacerbated by widespread mental health issues, severe food insecurity (50% acute vs. 37% moderate), and elevated homicide rates (13.35 vs. 1.35 per 100,000 inhabitants). As calls grow for France to return the independence ransoms extracted from Haiti, there remains limited data on how reparations could impact the country’s public health, community well-being, or effective implementation of healing programs. Between Spring and Fall 2023, we conducted 4 focus groups: 1st with Haitian men and women residing in the United States, a 2^nd^-with men in Haiti, a 3rd with women in Cap-Haïtien and Les Cayes, and a 4th with women in Cité Soleil. We conducted focus groups structured interview protocol, comprised of open-ended questions categorized into 4 thematic sections. These questions provided insights into participants’ perceptions on mental health, the daily challenges and barriers to access care, and community-based healing. Participants emphasized need for policies that address the social determinants of health, ensure safety and justice, and promote healthier workplace environments. They also advocated for mental health education aimed at reducing stigma, cultivating trust, and strengthening community support systems; with an emphasis on developing professional training, ethics, and sustainable long-term mental health services accessible for individuals of all ages. Haitian participants underscore the critical need to restore security, address the social determinants of health, and implement community-based mental health initiatives. We propose a biopsychosocial-ecological approach to guide reparations efforts. A targeted investment of $30 billion could yield substantial improvements in healthcare, mental health services, and public safety—contributing to increased life expectancy, reduced mortality rates, and decreased violence.

## Introduction

Haiti, over 220 years after its independence, continues to struggle with extreme poverty and impoverishment, a situation deeply rooted in historical injustices, anti-Black racism, a colonial legacy, and neocolonialism [1–3]. This includes the colonization by European powers, the genocide of the indigenous Taïno Arawak people, the slave trade, the enslavement of African ancestors, and the crippling economic impact of the reparations indemnity paid to France following the Haitian Revolution. According to eminent French economist, Thomas Piketty, this “double ransom of independence” of 90 million Gold Francs represented three years of Haitian production of 1825 and 300% of current Hait’s GDP or over $US 30 billion [4], deprived Haiti of much of its export revenues, favoring France and draining Haiti’s treasury [5] which severely limited investments in essential sectors like healthcare [4–7].

### Compelling Statistics Underpinning the Calls for Reparations and Restitutions for Haiti in 2025

Over 15 million men, women, and children were victims of the tragic transatlantic slave trade, which lasted approximately 400 years (United Nations, International Decade for People of African Descent; 2015–2024) and is considered one of the greatest genocides in history [8]. Prior to the 1791 revolution, three-quarters of a million captive Africans were dragged to colonial Haiti through the slave trade [9]. Combined with two centuries of enslavement and the ransoms of independence, the repercussions of the slave trade are profound and long-lasting on the mental and physical well-being of Haitians as well as on their political and economic stability, educational system, science, environment, spirituality, and more [10]. Two hundred years after the payment of this independence ransom was ordered, in 2025, Haiti and France present vastly different socio-economic and political realities. As indicated in Table 1, Haiti faces significant challenges in political stability and public health, while France, despite its own challenges, is more developed and stable [11–17]. However, in 2025 amidst the ongoing political crises and the growing demand for France to return the independence ransom to Haiti, there is limited data on how slavery reparations and the ransom restitutions could impact Haiti’s public health and community well-being or how healing programs could be effectively implemented.

**Table 1:** Overview of health disparities between Haiti and France in 2024 and projected health improvements in haiti through reparations-based investments.

Health Sector	Haiti (11.6 million inhabitants)	France (68,4 million)	Estimated Impact of Restitution ($30 Billion)
Infectious diseases outbreaks (introduced by the slave trade)	Anthrax, Cholera, Diphtheria, Dysentery, Influenza, Hepatitis B, Leprosy, Malaria, Measles, Parvovirus B19, Smallpox, Tetanus, Tuberculosis, Typhus, Yaws, Yellow Fever		Major reductions in infectious diseases like cholera and malaria with substantial investments in public health infrastructure.
Water and Sanitation	26% lacks access to an improved water source, with this figure rising to 41% among rural residents. 70% do not have access to an improved sanitation system, representing the lowest levels of access in the Western Hemisphere.	Universal access (99% of the population)	Advancements in water and sanitation infrastructure could raise access to improved water sources and sanitation systems to over 90%, leading to significant reductions in waterborne diseases and improved public health outcomes across urban and rural regions.
Life expectancy at birth (2023)	64.8 years	82.3 years	Could increase to 78–80 years over 10–15 years with $30 billion, assuming a 1.5% increase per $1 billion invested.
Life expectancy at birth (men)	61.7 years	79.4 years	Expected to increase to 74–75.5 years with targeted investments in male healthcare over a decade.
Life expectancy at birth (women)	67.3 years	85.1 years	Expected to rise to 80–82 years with focused investments in maternal and women’s healthcare services.
Infant mortality per 1,000 live births	47	3.1	Could decrease to approximately 10 per 1,000 live births with $8 billion invested in maternal and infant care.
Maternal mortality per 100,000 live births	480 (highest in the Caribbean)	8	Could reduce to 120 per 100,000 live births with $8 billion invested in maternal health programs.
Suicide rate (2018–2024) per 100,000 inhabitants	5.7 to 11.17	13.8	With better mental health services, the suicide rate could stabilize around 4–5 per 100,000 inhabitants.
Food insecurity	50% (5.4 million)	37%	Could decrease to 20–25% with investments in agriculture and food security programs, assuming a 25–30% reduction.
Prevalence of infectious diseases	Cholera, malaria, HIV/AIDS (highest in the Caribbean)	Seasonal flu, COVID-19	Major reductions expected in cholera and malaria. HIV prevalence could be reduced by 40–60% with public health investments.
Rate of Hypertension	27%	25%	Could decrease to 20–22% with improved healthcare access and early intervention programs.
Rate of Diabetes	8%	5%	Could reduce to 5–6% with investment in public health education and better healthcare access.
Rate of Hypercholesterolemia	18%	15%	Could reduce to 12–13% with enhanced healthcare services and public health campaigns.
Prostate cancer prevalence and mortality per 100,000 men	767 to 403 (one of the highest prostate cancer rates globally)	99–8	Mortality could decrease by 30–40% with $3–5 billion invested in cancer screening and treatment programs.
Cancer in women (Median age of diagnosis and mortality rate)	49 for Breast Cancer (BC), highest recorded death rate from cervical cancer globally.	63 for BC	A reduction in mortality of 20–30% is expected with $3–5 billion investment in cancer prevention and screening.
Gender-based violence (GBV)	Extremely high. 1.2 million women and girls in Haiti need GBV protection.	Moderate (rate unknown)	Could reduce by 40–50% through legal reforms, social services, and healthcare support with $2 billion allocated.
Femicide rate per 100,000 women	3.5	0.5	Could decrease by 50–60% with $2 billion invested in women’s protection and judicial reforms.
Availability of hospital beds per 1000 inhabitants	4.8	6	Could rise by 20–30% with investments in healthcare infrastructure and resources.
Universal health coverage	Non-existent	Yes, universal social security	Could establish universal healthcare covering 70–80% of the population within 10–15 years if $12 billion is invested.
Number of doctors and healthcare providers per 1,000 inhabitants	0.25	3.4	Could increase to 0.35–0.45 doctors per 1,000 inhabitants with $3 billion invested in medical education and healthcare infrastructure.
Leading causes of mortality	Infectious diseases, cardiovascular diseases, obstetric complications. The median age for both ischemic and hemorrhagic stroke is 10 years lower compared to other low- and middle-income countries (LMICs).	Cardiovascular diseases, cancers, respiratory diseases	Infectious disease mortality could reduce by 50–60%, and non-communicable diseases could emerge as leading causes of death with long-term healthcare investments.
Rate of Homicide & armed violence per 100,000 inhabitants	About 13.35	About 1.35	Could reduce by 40–50% over a decade with $3 billion invested in education, job creation, and social welfare programs.

#### A public health framework for haiti reparations and restitution.

Recent scholarship at the intersection of public health and reparative justice has laid critical groundwork for understanding how historical and structural violence contributes to health inequities among marginalized populations. Drawing on this body of work, we argue that any discussion of healing and generational trauma in Haiti must be informed by a reparative public health framework—one that centers the lived experiences of Haitian communities and acknowledges the enduring legacy of colonialism, slavery, and economic exploitation. Several studies have advanced this framework. Richardson et al. (2021) [18] demonstrate how reparative policies for Black American descendants of enslaved people could significantly reduce viral transmission and improve public health outcomes. Soled et al. (2021) [19] further argue for health reparations as an ethical imperative and a pathway to equity, emphasizing the moral and structural failures of existing health systems. Whittaker et al. (2025) [20] build on this by showing how racialized wealth gaps directly shape health disparities, suggesting reparations as a necessary intervention to redress these harms. Similarly, Bassett et al. (2020) [21] make the case for reparations as a public health priority, framing it as a practical and moral strategy for ending racial health disparities. Applying these insights to the Haitian context, we contend that reparations must not be understood solely as financial compensation or state-led acknowledgment of past injustices. Rather, they should encompass a broader, community-informed vision of healing that addresses current material conditions, promotes social and psychological restoration, and invests in public health infrastructure shaped by Haitian voices. Here, we employ a return-on-investment model, proposing preliminary projections for health improvements in Haiti through restitution-based investments totaling $30 billion (Table 1).

**Fig 1:**
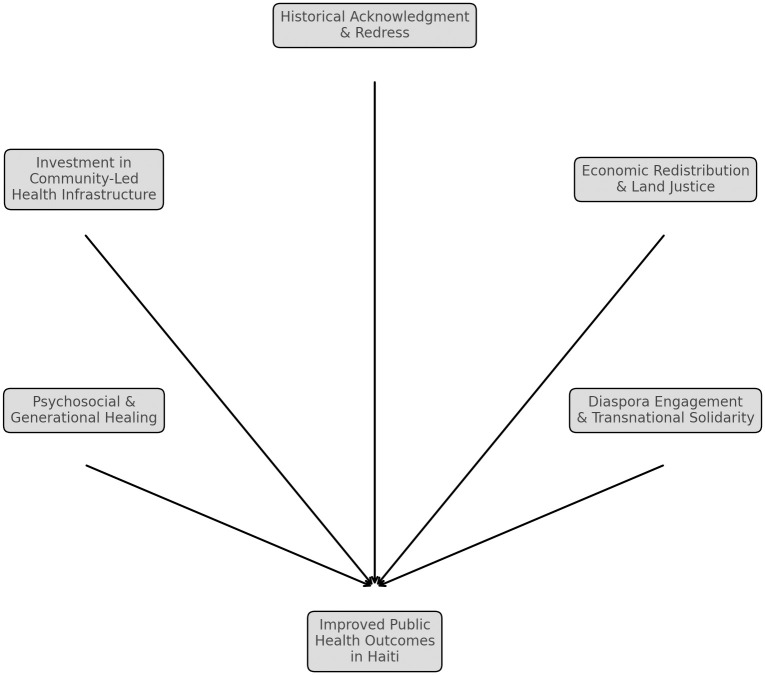
Pathways for reparations and restitution to improve public health in Haiti.

In May 2024, as part of the Haiti national reparations committee, we established a task force focused on “Historical/Multigenerational Traumas and Health Reparations.” The goal is to advance reflections and data from a biopsychosocial-ecological (BPSE) perspective on the public health impacts of historical and generational traumas, including the invasion of Ayiti, the *lands of high mountains* (Saint-Domingue) by Christopher Columbus in 1492, the enslavement of Indigenous and African ancestors, European colonization, and the ransoms Haiti were forced to pay to plantation owners and the State in France, Germany and the United States (U.S) [22–24]. Building on the National Academies of Sciences, Engineering, and Medicine, 2022 & Stineman [25] and Streim, we also aim to explore the modalities of reparations through a health-focused lens (Table 1, Fig 1).

### A biopsychosocial-ecological paradigm for reparations and Haitian healing

*“And power, money, and access to resources — good housing, better education, fair wages, safe workplaces, clean air, drinkable water, and healthier food — translate into good health.”.*

*Bassets and Galeo, 2020* [21]

The BPSE model, guided by Health Environmental Integration (HEI), promotes a comprehensive understanding of how illness, injury, limitations in activity, and restrictions in participation arise from the interaction between individuals and their environment. HEI acknowledges that complex, multilevel functional systems operate from the cellular level to an individual’s experience within their environment [22]. The HEI framework offers a holistic approach to understanding the generational traumas associated with slave trade, chattel enslavement, colonialism and human exploitation. In Haiti’s case, centuries of physical and psychological aggression and injuries stemming from race-based slavery and colonial oppression continue to affect the population today [10]. HEI identifies the social and physical environments as crucial in both causing and exacerbating diseases and disabilities. The impoverishment, social inequalities, and lack of healthcare access in Haiti, rooted in colonial exploitation, perpetuate health disparities (see Table – and Fig 1). A global reparations and restitutions movement should therefore go beyond a moral and symbolic recognition and the financial compensation to address these environmental factors that continue to harm the Haitian population.

The BPSE model also suggests that the very environments responsible for harm can become sources of healing. In the case of Haiti, reparations and restitutions from former colonizers such as France, Germany and the United States, should encompass the development of healthcare systems, access to cleaner air, drinking water, better schools, the restoration of cultural and social networks, and psychological interventions to address unhealed historical traumas. Furthermore, the model’s emphasis on integrating the body, mind, and societal connections promotes a broader understanding of healing—one that involves the restoration of dignity, social cohesion, safer neighborhood, and cultural identity. Future research could examine how the biopsycho-ecological model fosters interdisciplinary approaches, guiding the reparations movement in uniting efforts across various fields to achieve comprehensive healing for Haiti, and how this framework addresses both immediate health needs and the long-term restoration of societal well-being.

As the call for slavery reparations to Black populations in the African diaspora, including Haiti, is gaining momentum, our working group aims to explore a wide range of areas that could be tackled within the context of a transnational reparations movement in Haitian communities to promote multigenerational healing and social well-being [26]. In Table 1 and Fig 1, we illustrate this by leveraging a projected impact approach to estimate proportional health improvements based on financial investments in specific Haitian health sectors over time. We compare health disparities between Haiti and France in 2024, projecting improvements in Haiti with a $30 billion independence ransoms restitution investment. Haitian life expectancy could increase from 64.8 to 78–80 years, accompanied by significant reductions in infant and maternal mortality. Significant decreases in infectious diseases like cholera and HIV, along with improvements in chronic conditions such as hypertension and diabetes, are expected. Suicide rates, gender-based violence, and homicide could drop significantly with targeted investments. Healthcare infrastructure would expand, potentially establishing universal coverage for 70–80% of the population within 10–15 years (See Table 1 and Fig 1).

Our Haitian Well-Being Study, a pilot study project aiming mainly to identify psychosocial, neurological, genetic, sleep, physical health, socio-economic (SES), spiritual, and health behavior-related factors associated with mental well-being among a sample of Haitians in the U.S and Haiti, could offer a comprehensive scientific platform to nourish the public health framework of Haiti reparations and restitution movement. For example, through the Haitian Well-Being Study analysis conducted with Haitian men and women across Haiti and the U.S in 2023, we demonstrated the profound impact of Haiti’s deteriorating situation on individual and communal well-being [10]. Participants’ narratives underscore the severe health burden stemming from chronic exposure to socio-political instability, with reports of significant psychological distress and physical health complications such as hypertension, heart conditions, anxiety, substance abuse, suicidal ideations, sleep problems, depression, and post-traumatic stress disorders (PTSD). In this paper, we present the findings of the qualitative assessment that explores insights, guided by Haitian women, men, and caregivers, on how healing can be integrated into both personal lives and the broader Haitian community.

## Methods

### Data collection

We conducted four focus groups via Zoom with 28 participants from various demographics of the Haitian population, and representing broad perspectives with respect to themes under investigation; details regarding participant demographics and perspectives have been previously described [10]. Participants were located in the western (Port-au-Prince, Cite Soleil), southern (Cayes), northern (Cap-Haïtien), and Artibonite regions (Saint-Marc) of Haiti and the United States. We included Haitian immigrants from the United States, which is home to the most significant Haitian population outside of Haiti. As the crisis in Haiti has worsened over the past five years, more than 400,000 Haitian nationals have entered the United States. This figure includes individuals paroled into the country through a humanitarian program that also offers entry pathways to Cubans, Nicaraguans, and Venezuelans, in addition to those arriving through other immigration channels. As a result, the number of Haitian immigrants in the U.S. has grown substantially, reaching an estimated 852,000 in 2024, according to the Center for Immigration Studies. Both Haitians living in Haiti and Haitian immigrants or refugees residing in the United States have been directly and indirectly affected by the enduring consequences of colonialism, which have significantly contributed to health disparities and adverse health outcomes. They share a collective cultural memory and a unified Haitian identity, expressed through language, socioeconomic ties, and cultural practices aimed at stress reduction and improving overall quality of life. Additionally, many Haitians in the United States maintain strong transnational connections by providing financial support (e.g., remittances), material assistance (e.g., clothing), emotional support (e.g., long-distance communication), and educational resources (e.g., tuition or school supplies). These demographic realities underscore the transnational [10] nature of the Haitian crisis. The lived experiences of this crisis, shared by focus group participants, serve as the foundation for the insights presented in this article and affirm their authority to speak on healing and generational healing within Haitian communities.

This study was approved by the Institutional Review Board of the University of Miami Miller School of Medicine (IRB#:20211103). The team disseminated flyers to community-based organizations, health forums, social media, and texting groups. This ensured a broad and inclusive representation of voices. The research staff was trilingual in Haitian Creole, French, and English. The inclusion criteria specified individuals who were (a) aged 18 years or older, (b) either citizens of Haiti or identified as of Haitian descent and (c) proficient in English or Haitian Creole. Exclusion criteria excluded individuals (a) with cognitive impairments that might hinder survey completion or understanding of the study’s risks and benefits, (b) who did not identify as Haitian or of Haitian descent, and (c) who were not proficient in English or Haitian Creole.

### Inclusivity in global research

Additional information regarding the ethical, cultural, and scientific considerations specific to inclusivity in global research is included in the Supporting Information (S1 Checklist).

#### Procedures: Focus group.

Semi-structured interview guides were used for the focus groups. The focus group guide asked questions about suggestions for healing and moving forward. (See Table 2). The focus groups lasted 60–90 minutes. These sessions were audio recorded. An independent transcription and data management company, *Datagain* with trained experts in qualitative analysis, transcribed the focus groups. The focus group transcriptions were reviewed by the study’s principal investigator. The transcripts were de-identified and reviewed for accuracy. The transcripts were then translated from Haitian Creole to English by bilingual graduate-level study staff. The independent company analyzed the focus group data using thematic analysis.

**Table 2:** Focus group guide.

Domain	Questions
Suggestions for Healing and Moving Forward	What would you want to see in a treatment intervention?
	What would be helpful to you for your healing/well-being?
	What would be helpful to the Haitian communities for their healing/well-being?
	What suggestions do you have for moving forward?
	How do you think healing can be incorporated into your lives and in the Haitian community?

#### Data analysis.

The study employed thematic analysis to explore and interpret key conceptual and experiential patterns related to mental health as expressed by participants across various focus groups. This method was selected to systematically identify and analyze themes that addressed the study’s core research questions and overarching objectives. Participants were purposefully selected to reflect a diversity of perspectives within the Haitian community, including Haitian individuals residing in the United States and Haiti. Four distinct focus groups were conducted by study PI and lead author: one with Haitian men and women based in the U.S., another with men in Haiti, a third with women in Cap-Haïtien and Les Cayes, and a fourth with women in Cité Soleil. Guided by an intersectional framework, we remained attentive to differing needs, power dynamics, and the reality that women are generally more vulnerable to crises than men. With the increase in Gender-Based Violence (GBV) in Haiti, we anticipated that the topic might arise during the discussions and sought to create a space in which Haitian women felt safe to share their lived experiences of the Haitian crisis—a crisis largely shaped by male-dominated structures. In particular, we aimed to ensure that participants could speak openly about their heightened vulnerability to sexual violence and vision for healing in the current context of Haiti.

Each discussion was guided by a structured interview protocol comprising open-ended questions organized into four thematic sections. These questions were designed to elicit detailed insights into participants’ perceptions of mental health, challenges they face, barriers to accessing care, and community-based healing or coping strategies (See Table 2).

Prior to analysis, all focus group transcripts were de-identified by the Principal Investigator (PI) to protect participant confidentiality. A research team member then reviewed each transcript line by line, verifying and correcting the content as needed to ensure transcription accuracy. Graduate level and bilingual team members fluent in Haitian Kreyol and English translated the transcripts into English to facilitate comprehensive analysis.

The data were thematically coded using a combination of inductive and deductive approaches. The themes for each question or set of questions were compared and contrasted once combined into a master Excel spreadsheet and reviewed in the context of writing the report. If required, verbatim from the transcripts were re-coded on the spreadsheet. The phrasing of the theme statements in the report was then finalized to provide meaningful summary statements that addressed each specific question or overarching goal, where possible. The first phase was inductive. This was followed by a six-phase [27], deductive analysis by constantly comparing codes and the identification of themes. We conducted verification and legitimacy procedures by investigator triangulation, intercoding, reliability, rigor, and with in-depth descriptions [27,28] which included: (1) familiarization with the data, (2) generation of initial codes, (3) organization of codes into potential themes, (4) review and refinement of themes, (5) clear definition and naming of themes, and (6) final analysis and report production. A specialized codebook, developed specifically for this study, was used to guide the coding process. This codebook provided clear definitions and descriptions for each theme, ensuring consistent interpretation and alignment with participants’ lived experiences [28]. The coding process involved close examination of the content and, where necessary, re-coding of verbatim transcript excerpts within a centralized Excel spreadsheet [28].

To ensure analytical rigor, interrater reliability was assessed qualitatively, and collaborative reviews were conducted among team members to validate the consistency and accuracy of the thematic coding. Discrepancies and concerns regarding interrater reliability were addressed through team meetings, during which analysts collaboratively discussed divergent interpretations and outlined subsequent steps. Final themes and subthemes were determined only after the three analysts conducted a vote and reached unanimous consensus [28–31].

Themes that emerged across different focus groups and questions were systematically compared, contrasted, and refined [30]. The final wording of the thematic statements was developed to provide meaningful summaries that addressed both specific interview questions and the broader aims of the research. The PI adopted a narrative analysis [32] in communicating the findings, allowing for a coherent synthesis of the themes that highlighted key mental health issues, support mechanisms, and the broader social context experienced by the Haitian community.

### Ethics approval and consent to participate

Before data collection, the study protocol was reviewed and approved by the Institutional Review Board (IRB) of the University of Miami Miller School of Medicine (IRB#: 20211103) to ensure ethical compliance. The approval letter was issued by the University of Miami Human Subject Research Office (HSRO) in November 2022. Participants were recruited through the research team including the study P.I, a native of Haiti’s established networks in the US and Haiti using multiple approaches. Prior to participation, written informed consent was obtained from each individual. Following a screening process, participants provided informed consent online before taking part in focus group discussions. We enrolled participants between April 21st and November 4, 2023. Additional information regarding the ethical, cultural, and scientific considerations specific to inclusiveness in global health research is included in the Supporting Information (See S1 Checklist).

#### Positionality statement.

The research team included Haitian and non-Haitian female and male scholars with interdisciplinary backgrounds in public health, psychology, and social justice. Team members with lived experiences in Haitian communities and fluency in Haitian Kreyol contributed to culturally grounded data collection and interpretation. Recognizing our varied positionalities, having advanced degrees ranging from Master’s, PhDs, and Medical Degrees- brought an in-depth critical and self-reflection, that brings an increased awareness to our privilege, status, and power. We drew upon our experiences of struggle, racism, discrimination, inequality, and health disparities’ being in the Caribbean diaspora – while pursuing advanced degrees. According to Horsburg, critical reflection enhances the rigor of the study and ethics since ‘The researcher is intimately involved in both the process and product of the research enterprise, it is necessary for the reader to evaluate the extent to which an author identifies and explicates their involvement and its potential or actual effect upon the findings [33]. We engaged in ongoing reflexive practices to mitigate potential biases and ensure credibility and validity by accounting for researchers ideologies, knowledge, and knowledge production, through trustworthiness and honor of participants’ voices and lived experiences [34–38].

Critical self-reflection and introspection offers many benefits regarding the research process, researcher representation, critical analysis of power dynamics, methods, interpretation, connecting with subjects, boundaries, logistics, requirements, and unexpected matters in conducting research at any given situation [39–41]. Critical self-reflection as researchers, scientists, professors, at elite universities comes with a sense of privilege, however we as authors have experienced directly or indirectly the long standing historical colonial consequences of discrimination and oppression through structural inequalities and inequities. It is understood that although we have advance careers, there are still individuals’ part of the Caribbean diaspora, specifically Haitians, unfortunately who do not have similar opportunities and access to resources to improve their quality of life.

## Results

Each focus group consisted of 5–10 individuals. The cohort comprised 28 individuals overall, with 20 women and eight men (Mean age = 29.5, SD = 9.8). One session included women from Cite Soleil only, one session included Haiti-based men, another session included women from various departments of Haiti, and the last one was composed of U.S.-based Haitian Americans and immigrants.

[10] Our discussions on improving mental health support highlighted the importance of addressing social determinants of health, such as housing, financial strain, unemployment, education, and ensuring societal security through justice and crime prevention. Participants emphasized the need for healthy workplace policies, mental health education to reduce stigma and build trust, and comprehensive professional training guided by ethical standards. A recurring theme was the necessity of building sustainable, long-term accessible mental health services to individuals across all age groups. Direct quotes from the participants in English and Haitian Creole are provided in Table 3.

**Table 3:** Multigenerational healing and public health recommendations in their own voices.

Factors	Direct Quotes	English Translation
Social Determinants of Health Stressors	*“M ta renmen pou n ka jwenn swen. Epi, pou pwoblèm ensekirite a rezoud, pou lavi chè a desann. Paran yo ka voye timoun yo lekòl, paske pa gen travay. Lavi a di. Gen anpil fwa yo pa ka voye pitit yo lekòl. Gen anpil paran ki oblije chita lakay yo, epi yo pa ka soti, m ta renmen bagay sa yo rezoud pou ta ka genyen yon [inaudible]. Paske nan moman la ann ayiti, fanmi yo [inaudible], preske [inaudible] lopital yo fèmen, tout doktè yo fin pati. Se komsi, nou menm, nou jis lage poukont nou, [inaudible] l ap pati. Nou jis lage poukont nou, e nou ta renmen sa rezoud. Paske nou menm, peyi a se yon bon peyi e nou ta renmen rèt lakay nou, pou n travay, pou tout bagay vin se menm nan malman. Mwen menm panse sa ta sipoze rezoud pou ke nou ka geri.*	“I would like for us to be able to access care. And for the insecurity problem to be resolved, and for the high cost of living to go down. Parents could send their children to school, because there is no work. Life is hard. Many times they cannot send their children to school. Many parents are forced to stay at home and cannot go out—I would like these things to be resolved so that there could be a [inaudible]. Because right now in Haiti, families are [inaudible], almost [inaudible], hospitals are closed, all the doctors have left. It’s as if we have just been abandoned, [inaudible] is leaving. We are just left on our own, and we would like that to change. Because we, our country is a good country, and we would like to stay home, to work, so that everything could become stable again. I personally believe that needs to be resolved so that we can heal.”
Security Issues	*“Pou kominote a geri nèt, pou l kapab geri, vrèmanvrè pou n jwenn gerizon nou, se ensekirite a, ki pou pa la ankò. Menmsi se pa a 100%. Menm a 70 a 80%. Si nou ta gen yon sekirite ki bokou stab pou nou. Sa te ka ede nou.* *Paske, lè nou gade nan tout kwen yo, vrèmanvrè, lè nou sibi depresyon nou genyen yo, se pwoblèm sekirite ki fè sa.” (Female, Cité Soleil).*	“For the community to heal completely, to truly find our healing, it’s the security that is no longer here. Even if it’s not 100%, even at 70 to 80%. If we had a very stable security for us, that could help us.” (Female, Cite Soleil)
Mental Health Services and Education	*“Se gerizon au cas par cas, jesyon emosyon jan kamarad lan dil la, mwen dakò, jesyon strès epi pafwa terapi au niveau konpòtemantal.” (Male, Port-au-Prince)* *“Si n ap pale de gerizon, tretman, dabò fòk moun nan admèt ke li malad, donk se vre nou menm nou plis kwè maladi a li fizik donk nou pa vreman fokis sou sikoloji an.” (Female, Les Cayes/Cap-Haitien)*	“It’s case-by-case healing, emotion management as the friend said, I agree, stress management, and sometimes behavioral therapy.” *(Male, 38)* “If we are talking about healing, treatment, first the person must admit that he is sick, so it is true that we believe the disease is physical, so we do not really focus on the psychological.” *(Female, 42)*
Culturally Tailored Interventions	“Sante mantal ki teni kont de aspè kiltirèl la.”	“Mental health that takes into account the cultural aspect.”
Multigenerational Long-term Services	*Yon pwogram ki vreman pa yon pwogram efemè, yon pwogram dirab sou kelke ane k ap panche sou pote èd non selman a jèn fi paske m wè etid lan plis baze kesyon jèn fanm, jenòm, nou selman pote sipò an sante mantal pou jèn yo men sitou pou timoun yo. Sa vrèman ka sa msansib pou li anpil. E sa fè nan tout, he, nan tout sijesyon ke mwen ka pote, eske gen posibilite nan menm pwogram sa, nan swivi k ap gen pou fet, eske kapab gen yon pati ki pran ka sante mantal pou timoun yo? Pa selman jèn, se vre nou menm jèn nou gen bezwen l anpil men timoun yo ap viv sitiyasyon non selman ensekirite e pwoblèm katastròf natirèl, pwoblèm ensètitid pou demen, timoun yo ap viv li pi mal ke moun ki adilt. Non selman pou pote yon chanjman, yon amelyorasyon pou pote yon gerizon an pwofondè. Se pa yon pwogram k ap la inikman swa sou baz yon jou oubyen 2 jounen e de sansibilizasyon aktivite avèk jèn epi sa fini la. Eske ap gen posibilite pou pwogram nan kreye yon, yon, yon posibilite pou kapab pote yon solisyon an pwofondè pou sante mantal, timoun, jèn, granmou an Ayiti*. (Female, Les Cayes)	“My expectation, especially after a peer study, is a program that is really not an ephemeral program, but a sustainable program over several years that will focus on bringing help not only to young women because I see the study is more based on the question of young women, young men, we only bring mental health support to young people but especially to children.” “It is not a unique program either on the basis of one day or 2 days and two awareness activities with youth and that ends there.”

### Social determinants of health stressors

Participants noted that there was a lack of employment and hence money, which would need to be dealt with in order to promote healing and well-being. We need employment to survive/*Nou bezwen gen travay pou nou ka siviv* stated a female participant from Cite Soleil. This lack of employment leads to an inability to send their children to school. Another participant from Cité Soleil noted, “Parents cannot send their children to school because there is no work.” Life is hard/*“Paran yo pa ka voye timoun yo lekòl, paske pa gen travay. Lavi a di.”* In addition, these factors are amplified by lack of access to health care. That participant noted “the hospitals are closed, all the doctors have fled the country*/ “lopital yo fèmen, tout doktè yo fin pati*.”

### Security issues

Participants noted a strong need for greater security for Haitians to heal as well. One participant noted “For the community to heal completely, to be able to heal, to truly find our healing, it’s the security, that is no longer here. Even if it’s not 100%. Even at 70 to 80%. If we had a very stable security for us. That could help us/ *Pou kominote a geri nèt, pou l kapab geri, vrèmanvre pou n jwenn gerizon nou, se ensekirite a, ki pou pa la ankò. Menmsi se pa a 100%. Menm a 70 a 80%. Si nou ta gen yon sekirite ki bokou stab pou nou. Sa te ka ede nou*.” There was also a connection between a lack of security and depression. Furthermore, the participant noted “Because when we look at all the corners, truthfully, when we suffer the depression that we have, it’s the safety issues that cause it/ Paske, lè nou gade nan tout kwen yo, vrèmanvrè, lè nou sibi depresyon nou genyen yo, se pwoblèm sekirite ki fè sa.”

### Mental health services and education

In order to heal, participants also felt there was a need for both mental health services and education. A male participant noted, “It’s case-by-case healing, emotion management as the friend said, I agree, stress management and sometimes behavioral therapy/*Se gerizon au cas par cas, jesyon emosyon jan kamarad lan dil la, mwen dakò, jesyon strès epi pafwa terapi onivo konpòtemantal*.” A female participant stated “If we are talking about healing, treatment, first the person must admit that he/she is sick, so it is true that we believe that the disease is physical, so we do not really focus on the psychological/*Si n ap pale de gerizon, tretman, dabò fòk moun nan admèt ke li malad, donk se vre nou menm nou plis kwè maladi a li fizik donk nou pa vreman fokis sou sikoloji an*.” In addition, she noted “the education session would be really, really important, especially for me/*seyans edikasyon an t ap vreman, vreman enpòtan sitou mwen menm*.”

### Training community leaders

One female participant from Les Cayes mentioned training.

I would suggest that we train a group of people, a group of young people, and they will do the same thing that you are doing in the community, while you train them, they have training, they know how to do it and in return they will go to the community to take information from people to see what people think about mental health.”

### Long-term services for all generations and genders

There was also a need beyond this study for long-term services for children, youth, and the elderly to aid with healing. A female participant noted “My expectation, especially after this kind of study, is a program that is really not an ephemeral program, a sustainable program over several years that will focus on bringing help not only to young women because I see the study is more based on the question of young women, young men, you can bring mental health support to young people but especially to children/*Atant mwen sitou aprè yon etid parèy se yon, yon pwogram ki vreman pa yon pwogram efemè, yon pwogram dirab sou kelke ane k ap panche sou pote èd non selman a jèn fi paske m wè etid lan plis baze hé kesyon jèn fanm, jenòm, nou selman pote sipò an sante mantal pou jèn yo men sitou pou timoun yo*.” In addition, she describes a program to help with healing, “It is not a unique program either on the basis of one day or 2 days and two awareness activities with youth and that ends there/ *Se pa yon pwogram k ap la inikman swa sou baz yon jou oubyen 2 jounen e de sansibilizasyon aktivite avèk jèn epi sa fini la.”* The program would need to be a long-term solution rather than just for a few days. Furthermore, she asked about “, a possibility to be able to bring an in-depth solution for the mental health of children, youth, and the elderly in Haiti/ *yon posibilite pou kapab pote yon solisyon an pwofondè pou sante mantal, timoun, jèn, granmou an Ayiti*.”

## Discussion

The primary objective of this study was to provide on-the-ground insights into the urgent need for a multigenerational healing movement within Haitian communities, particularly in the context of a state dismantling [10] and the increasing calls for reparations and restitution for slavery. This study utilized the BPSE model, guided by the HEI framework that comprises of comparing current health disparities between Haiti and its former colonial power, France, with a focus on life expectancy, morbidity, and healthcare infrastructure. Additionally, a return-on-investment model was applied to project potential health improvements based on a proposed $30 billion investment in healthcare, public health, and social services. Our focus groups insights align with an April 2024 survey conducted by the Haitian women-led coalition “Rasanble Pou Ayiti,” which gathered responses from over 1,000 participants across Haiti’s ten departments, revealing that psychological support is among the population’s top priorities, alongside security, education, and economic stability [22]. Based on this work, in the Haitian context addressing social determinants of health, establishing mental health services and interventions, improving mental health education, training community leaders and having long-term services are all key elements identified through the use of the HEI framework to consider when formulating plans for reparations, restitution, and generational healing.

### Social determinants of health

This study argues that social determinants of health such as economic instability, lack of education, poor health care access, and security issues must all be addressed for the Haitian community to heal. Extensive literature has examined the role of social determinants of health on poor health outcomes. It has been noted that reparations for Black individuals may help in improving health outcomes [20]. Just as reparations for Black individuals in the US, it may also benefit those living in Haiti.

### Mental health services and interventions

Focus group participants acknowledged the need for diverse modes of mental health services and programming, including both individualized and collective strategies. Individualized mental health support requires opportunities for personalized, “case-by-case” therapeutic relationships between the provider and the patient. In addition to traditional diagnostic and therapeutic mental health services, participants specifically recognized the importance of strategies that include emotional management, stress management, and behavioral therapy. Research supporting emotion management programs has shown promising results in patients with schizophrenia spectrum disorders, including improved emotion recognition and expression, enhanced connectivity in the amygdala and prefrontal cortex, and overall better quality of life [42–45].

Stress management and coping interventions are gaining attention in immigrant communities, although Haitian communities have not been studied as extensively as other immigrant populations [46–50]. Similarly, behavioral therapies have shown promise in treating PTSD, depression, and related mental health disorders in underserved immigrant populations, though the literature focusing on Haitians is limited [51–56]. Our findings suggest that members of our cohort are aware of the need for individualized mental health services and specific strategies within Haitian communities, which could benefit many community members as they have benefited other international and immigrant communities [10].

*“Nou konnen nou ka konte sou lòt” (We are aware that we can count on one another)*

In addition to individualized services, participants highlighted the need for collective and community healing and the creation of a supportive environment. Beyond individual needs for personalized approaches, there is a recognized collective need for mental health services support that addresses Haitian communities’ unique challenges and needs. This finding may reflect a core Haitian cultural value of solidarity, often described as *tèt ansanm*, literally translated as “heads together.” *Tèt ansanm* has been recognized as a vital, collective source of resilience in the face of struggles common to Haitian communities globally [57,58]. The latter value was well illustrated by a Haiti-based male participant: *Nou konnen nou ka konte sou lòt e se de faktè de pwoteksyon e kwayans nou tou, e pafwa li ka menm yon espwa.* These mental health services and interventions however would require money and as such reparations could play a significant role.

### Mental health education

The need for education and culturally responsive training about mental health and illness was specifically cited as key components of effective mental health care interventions. Focus group participants recognized that some degree of insight and awareness into one’s mental health is required to begin the journey to seek mental health support. Conversely, the stigmatization of mental illness may result from a lack of education about mental health and disease, and educational interventions may provide a means of decreasing stigma-related barriers to seeking mental health care [59–61]. More broadly, access to education and level of education have been linked with mental health care-seeking behavior and access to mental health services, especially in low- and middle-income countries like Haiti and comparable socially disadvantaged populations [61,62].

### Training community leaders

Incorporating the suggestions from the focus groups into a comprehensive healing program would yield several benefits, such as reducing the stigma surrounding mental health in Haiti, increasing awareness and understanding, and equipping community members with the skills necessary to address their unique needs. Strengthening support through the use of community leaders would nurture collective healing and could contribute to improved mental health outcomes for the Haitian population. A crucial question to consider is how this large-scale mental health initiative can align with and contribute to the public health approach to Haiti’s reparations and restitution movement, while also ensuring its sustainability.

### Long-term services for all generations and genders

Culturally adapted approaches have been shown to be more effective and sustainable over time. Culturally adapted mental health care approaches were also identified as important for effective mental health support. Indeed, group therapy and related collective approaches may be more conducive to cultural adaptation in Haitian populations, given the socio-economic and cultural constraints. The limitations of individualized mental health paradigms and the potential value of group or collective mental health approaches have been described in civilian populations affected by war and conflict [63–65]. Moreover, the sustainability of all forms of mental health services, including long-term support for vulnerable populations such as youth and the elderly, was also highlighted as an important need in our focus groups. Collectively, these findings underscore the emergency to prioritize health equity, sustainability, personalized and collective care, and culturally adapted interventions for mental health support and trauma-focused therapy among our Haitian participants. These could all be supported by an influx of additional financial resources.

Overall, this study highlights the urgent need for a multigenerational healing movement in Haitian communities, linking historical trauma to current health disparities. The BPSE model, grounded in Health Environmental Integration, calls for reparations that include mental health services, healthcare infrastructure, and culturally adapted interventions. Focus group findings emphasize both individualized care and collective healing rooted in Haitian values like *tèt ansanm*. A $30 billion investment could significantly improve life expectancy and reduce disease burdens. Such reparative efforts must integrate education, sustainability, and public health to achieve long-term societal well-being.

### Limitations of the study

Our study possesses several strengths, particularly in its methodological rigor related to interpretative analysis and coder variability. The inclusion of community members and leaders in the research process provided a broader perspective on barriers to research participation. Moreover, participants contributed valuable insights that informed the development of strategies aimed at improving recruitment efforts. However, this qualitative study also has several limitations that warrant consideration. For instance, the representativeness of the sample is limited, as participants may not fully capture the diversity of experiences within the broader Haitian population. Furthermore, conducting focus groups virtually presented challenges related to participant engagement, technological connectivity, digital literacy, and communication dynamics. Future research should prioritize the inclusion of a more demographically and geographically representative sample. Employing quantitative methods, or a mixed-methods approach combining both quantitative and qualitative techniques, may offer a more comprehensive understanding of mental health stigma, depression, stress, and anxiety within Haitian diasporic communities. In addition, although this study provides a framework with key elements more data is needed regarding how reparations can be included in public health programs.

## Conclusion

This study contributes to a growing body of literature that calls for reparative action across the Caribbean diaspora, where the long-term effects of slavery, colonial exploitation, and socio-economic disenfranchisement continue to undermine health and well-being. Our findings support the adoption of a biopsychosocial-ecological model guided by the HEI framework to inform reparations and investment strategies. Our qualitative study explores the enduring consequences of colonialism that have contributed to significant health disparities among Haitian populations. These disparities are evident across multiple domains, including reduced life expectancy, elevated infant and maternal mortality rates, increased prevalence of mental health conditions, food insecurity, and high rates of violence. Participants highlighted the urgent need for policies that address the social determinants of health, promote public safety and justice, and improve working conditions. Central to their recommendations were calls for mental health education to reduce stigma, the cultivation of trust through community engagement, and the development of sustainable, ethically guided mental health services accessible across all age groups. Multifaceted reparations and generational healing, the way that Haitians, including participants from the Haitian Well-Being Study, envision them must be at the center of any public health intervention among Haitians, both in the country, within the diaspora, and larger Caribbean diaspora. Reparations and generational healing are vital ideas that are often ignored (and arguably, there may never be global health equity without either). Our study helps move the field closer to realizing the importance of these concepts.

## Supporting information

S1 Checklist Inclusivity in global research. (DOCX)
