# Supplementary material for: A public health framework for reparations and generational healing in Haiti
Source: PLOS Glob Public Health. 2025 Nov 4;5(11):e0004133. doi: 10.1371/journal.pgph.0004133 (PMC12585046; doi:10.1371/journal.pgph.0004133)
Supplement: S1 Checklist — (DOCX) [file pgph.0004133.s001.docx]

Inclusivity in global research

PLOS’ policy on inclusivity in global research aims to improve transparency in the reporting of research performed outside of researchers’ own country or community and ensures that PLOS publications reporting global research adhere to high standards for research ethics and authorship. Authors of relevant research articles may be asked to complete the questionnaire below, which outlines ethical, cultural, and scientific considerations specific to inclusivity in global research. This questionnaire may be requested when researchers have travelled to a different country to conduct research, if research uses samples collected in another country, research with Indigenous populations or their lands, or if research is on cultural artefacts. Researchers travelling to another country solely to use laboratory equipment will not normally be required to complete the questionnaire. However, the questionnaire can be requested at the journal’s discretion for any submission – if you have been requested to complete this questionnaire by the PLOS journal you submitted to, please do so.

Please complete the questionnaire below and include this as a Supporting Information file with your manuscript. Note that if your paper is accepted for publication, this checklist will be published with your article in the supporting information files. Please ensure that you reference the checklist in the main body of your manuscript. We suggest adding a subsection ‘Inclusivity in global research’ to your Methods section and adding the following sentence: “Additional information regarding the ethical, cultural, and scientific considerations specific to inclusivity in global research is included in the Supporting Information (SX Checklist)”

The questions have been designed to be applicable to a wide range of study types, and there are subsections for both human subjects research and non-human subjects research. If any of the questions are not relevant to your research please mark them as “N/A” as appropriate.

**Ethical considerations, permits and authorship**

*This section is applicable to all research types.*

Provide details as to who granted permissions and/or consent for the study to take place in the Methods section of your manuscript. This should include the names of **all** ethics boards, governmental organizations, community leaders or other bodies that provided approval for the study. If individuals provided approval refer to these people by their role or title but do not list their name(s).

Reported on page number: 13

If there were any deviations from the study protocol after approval was obtained please provide details of these changes in the Methods section of your manuscript.
Did this study involve local collaborators that are residents of the country where the research was conducted or members of the community studied? If you do not have any authors from said communities, please provide an explanation for this below.

Reported on page number: N/A

The Principal Investigator of the main study, Dr. Judite Blanc, is a Haitian-born psychologist based in the United States with strong affiliations to local Haitian organizations, including the State University of Haiti, the Psychological Association of Haiti, Zanmi Lasante (Partners In Health), and the Fontaine Foundation. The study flyer was distributed throughout this network. A portion of the focus group participants were recruited through the Fontaine Foundation in Cité Soleil, with support from the leadership of these organizations. Their contributions are recognized in the Acknowledgments section of the manuscript.

Everyone listed as an author should meet PLOS’ criteria for authorship and all individuals who meet these criteria should be included in the author byline, rather than the acknowledgements. For further information please see the journal’s Authorship Policy.

**Human subjects research (e.g. health research, medical research, cross-cultural psychology)**

Did you obtain written informed consent from a representative of the local community or region before the research took place? How did you establish who speaks for the community? Details of written informed consent obtained from study participants should be reported separately in the Methods section of your manuscript.

Dr. Judite Blanc is a Haitian-born researcher, and the two co-authors of this paper are Haitian Americans. This study was approved by the IRB at the University of Miami. All participants provided written consent in Haitian Creole before joining the focus group session.

How did members of the local community provide input on the aims of the research investigation, its methodology, and its anticipated outcome(s)?

The goal of the focus group was to gather insights from Haiti-based individuals, as well as U.S.-based Haitians and Haitian Americans, to inform the most appropriate methodological approach for the quantitative phase of the Haitian Wellbeing Study—a larger, 10-year research initiative.

When engaging with the local community, how did you ensure that the informed consent documents and other materials could be understood by local stakeholders?

The study's informed consent was written in lay language, translated into Haitian Creole, and reviewed by a Haitian linguist. Additionally, the enrollment process was carried out by Haitian-born study staff.

Will the findings of the research be made available in an understandable format to stakeholders in the community where the study was conducted (e.g. via a presentation, summary report, copies of publications, etc.)? Please provide details of how this will be achieved.

We plan to share the findings in lay language through Haitian media, during community town halls, and via short video clips distributed on social media platforms.

**Non-human subjects research using specimens/ animals collected as part of the study, or those housed in archival collections. Examples include archaeology, paleontology, botany and zoology.**

Did the permission you obtained from a local authority to perform the study include an agreement on access to outputs and benefit sharing? This may include procedures to enable fair distribution of the benefits and resources arising from the research performed. Please include any details of Prior Informed Consent and Benefit Sharing Agreements obtained. These may be required by field-specific regulations, for example the Convention on Biological Diversity (CBD) and the associated Nagoya Protocol.

N/A

If the material used in your study was imported, please A) provide the year it was imported and B) indicate whether permits were obtained to import/export the materials used, C) provide details of any permits obtained. If this information is not available, please indicate this.

N/A

If you used archival specimens, please state how the material used in your study was acquired by the institute it is held in and provide details of any permits obtained for the original excavations/ sample collection. If this information is not available, please indicate this.

N/A

How was the potential cultural significance of the materials collected in your study to local communities considered in your research design? Were Indigenous peoples and/or local researchers and institutions involved with archaeological excavations / collection of specimens? If so, please provide a description of their involvement.

The potential cultural significance of the materials collected in this study was carefully considered throughout the research design process. Recognizing the deep historical and cultural context of Haitian communities, particularly in relation to collective trauma, healing traditions, and mental health, the study was grounded in culturally responsive methodologies. Research tools, including interview guides and consent forms, were developed in collaboration with Haitian cultural advisors and translated into Haitian Creole to ensure accessibility and cultural relevance.

While this study did not involve archaeological excavations or the collection of physical specimens, it prioritized the ethical handling of sensitive narratives and community knowledge. The research team included Haitian-born scholars and practitioners, and the data collection process was led by Haitian researchers with lived experience in the communities being studied. Local institutions, including community-based organizations and advocacy groups, were consulted during both the design and implementation phases to ensure alignment with community values and needs.

Their involvement was instrumental in shaping the study's ethical framework, ensuring respectful engagement, and promoting reciprocity in the research process. This collaboration also supported the identification of themes that are culturally significant and relevant to ongoing conversations about mental health, reparations, and community resilience in Haiti.

If your manuscript includes photographs of human remains please indicate whether authors obtained permission from descendants or affiliated cultural communities to do so.

N/A
